# Possible Changes and Trends in Non-COVID-19 Vaccine-Prescribing Patterns before and during COVID-19 Pandemic

**DOI:** 10.3390/vaccines12060582

**Published:** 2024-05-27

**Authors:** Shirie van Rooyen, Martie Lubbe, Irma Kotze, Nkengafac Villyen Motaze

**Affiliations:** The Faculty of Health Sciences, School of Pharmacy, North-West University, Potchefstroom 2531, South Africa

**Keywords:** coronavirus disease, COVID-19, prescribing patterns, vaccines, non-COVID vaccines, medicine claims data, South Africa

## Abstract

Due to the COVID-19 pandemic, many children missed their routine vaccinations globally. There is insufficient evidence on the trends in vaccination coverage in the private healthcare sector in South Africa. This study explored the changes in childhood vaccination patterns (non-COVID vaccines) in the private healthcare sector in South Africa using medicine claim data. Using the information on medication claims from a South African pharmaceutical benefit management (PBM) company, we performed a quantitative cross-sectional analysis comparing the period before (2018–2019) and during the COVID-19 pandemic (2020–2021). All patients who made claims within the study period were included. This study included 67,830 children aged two years and younger. In particular, from 2018 to 2021, boys (52%) outnumbered girls (48%). Pharmacists consistently held the predominant prescriber role before and during the COVID-19 pandemic. The proportion of children receiving non-COVID-19 vaccines was higher before the pandemic (60%) than during the pandemic (55%). Furthermore, there was a notable decline of 5% in measles vaccination rates during the children’s first year of life, while a notable increase was observed for measles (5%), hepatitis A (7.7%), and the pentavalent vaccine (5%) during the second year of life. Governments and private healthcare providers must take action to enhance vaccination coverage rates for children in their first year of life to prevent a resurgence of vaccine-preventable diseases. The results obtained in this study underscore the significance of implementing vaccine catch-up campaigns to address missed vaccination opportunities arising from the impact of the COVID-19 pandemic. Moreover, pharmacists emerged as the predominant healthcare providers responsible for administering vaccinations within the private healthcare sector in South Africa, both prior and during the COVID-19 pandemic. Their pivotal role in the vaccination process warrants due recognition and should not be underestimated.

## 1. Introduction

Vaccines prevent more than four million deaths worldwide annually, and this makes vaccination a very effective and affordable public health intervention [1]. Vaccines protect against diseases of public health importance and connect individuals with healthcare systems, thereby providing the opportunity for delivering other fundamental primary healthcare services [2]. Therefore, ensuring vaccine access is one of the crucial measures to achieve universal health coverage (UHC) [2].

Coronavirus disease (COVID-19) is characterised as a highly infectious viral disease caused by the SARS-CoV-2 virus, as stated by the World Health Organization (WHO) [3]. On 13 January 2023, the WHO reported a total of 661,545,258 confirmed cases of COVID-19 and a staggering 6,700,519 deaths worldwide since the onset of the pandemic, delineating the profound impact of this pandemic on a global scale [4]. The COVID-19 pandemic has had significant and diverse influences on health systems worldwide, influencing multiple aspects of healthcare provision, workforce capability, and infrastructure. The disruption of routine healthcare services, including screenings, elective procedures, and nonurgent health appointments, were repercussions of the COVID-19 pandemic on healthcare systems worldwide. Interruptions in routine childhood vaccination programs in Southeast Asia and the Western Pacific World Health Organization (WHO) regions were well documented [5]. A global decrease of 7.9% was observed in childhood immunisations of the third dose of the Diphtheria-Tetanus-Poliomyelitis vaccine (DTP3) and the first dose of the measles-containing vaccine (MCV1) [5,6,7]. The most pronounced annual repercussions were observed in North Africa, the Middle East, South Asia, as well as in Latin America and the Caribbean. On the contrary, sub-Saharan Africa witnessed the most modest annual declines in vaccine coverage, characterised by a relatively undisturbed operational landscape throughout the year [3,4,5,6]. In Southeast Asia and Oceania, monthly dosages of the DTP3 and MCV1 vaccine were consistently administered at or exceeded anticipated levels during the latter half of 2020 [4,5].

Before the onset of the COVID-19 pandemic, vaccination coverage figures decreased globally, especially in 2018 and 2019 [5,6,7,8]. A systematic review conducted in the African context pertaining to challenges and threats encountered in childhood immunisations determined that several factors contributed to the sustained reduction in childhood vaccination rates. These included maternal education levels, political instability, dependence on foreign aid for vaccine procurement and distribution, and religious and economic factors [9].

Nonetheless, it is imperative to underscore that, to date, the most substantial impacts on childhood immunisation have been attributed to the COVID-19 pandemic [10]. According to a benefit–risk analysis undertaken in Africa, routine childhood immunization during the pandemic could have saved 84 child fatalities for every excess COVID-19 death linked to SARS-CoV-2 infections contracted during usual vaccination clinic visits [11].

The observed disruption in routine vaccination schedules further elevated the risk of vaccine-preventable disease outbreaks such as of measles, polio, diphtheria, and pertussis, especially in low- and middle-income countries. Lower- and middle-income regions already carry the burden of lower vaccination rates, underimmunised children, missed communities, and zero-dose children [12,13,14].

In South Africa, there was a measles outbreak in 2023 [15]. According to recent data from the United Nations Children’s Fund (UNICEF), South Africans’ trust in childhood immunisations has declined by 30%, and one in five children is underimmunised due to increased vaccine hesitation during COVID-19 [15]. Vaccine hesitancy has been linked to decreased vaccination coverage in other parts of the world. A longitudinal analysis of vaccination coverage in Brazil unveiled a noteworthy correlation between heightened online inquiries for antivaccine content and a concomitant decline in immunisation coverage rates [16].

According to UNICEF’s annual statistics, 67 million children worldwide had missed one or more vaccines over the previous three years [15]. More than 30 million African children under five still contract vaccine-preventable diseases each year, leading to over half a million deaths [15].

South Africa has a dualistic healthcare system, meaning a public healthcare system and a private healthcare system [17,18]. The private healthcare system in South Africa mainly consists of medical aid, and the remaining involves out-of-pocket payments [19]. As of 2023, 16.1% of the South African population were members of medical aid schemes [20]. Both the public and private healthcare systems in South Africa offer immunization services to children. Immunizations are provided at government clinics for free in the public health sector. Private nursing clinics, hospital baby clinics, and pharmacy clinics can all provide immunizations to children in the private healthcare sector. Pharmacists dispense vaccines and hence serve as prescribers at the majority of vaccination providers in the private healthcare sector, and nurses administer the vaccinations. 

To the best of our knowledge, there are no empirical data documenting the alterations in vaccination rates within South Africa’s private healthcare sector before and amidst the COVID-19 pandemic. Exploring the changes in vaccination patterns can be valuable for strengthening healthcare systems and increasing vaccine coverage in our most vulnerable populations. Therefore, this study aimed to explore the changes in vaccination patterns in the private healthcare sector in South Africa before (2018, 2019) and during (2020, 2021) the COVID-19 pandemic.

## 2. Materials and Methods

### 2.1. Study Design and Data Sources

A quantitative, analytical, cross-sectional design was used for this investigation. A South African pharmaceutical benefit management (PBM) company provided data on medical claims for a four-year period between 1 January 2018 and 31 December 2021. This PBM company is one of the largest computerised claims-processing and PMB providers for medical aid (health insurance) companies in South Africa, with over 1.8 million beneficiaries. Children aged two years and below for whom a claim was submitted for a vaccine that was part of the childhood immunization schedule (henceforth referred to as non-COVID-19 vaccine) were included in the analysis. The years 2018 and 2019 are referred to as the “before” phase, while 2020 and 2021 are referred to as the “during” phase.

Encrypted medical scheme numbers, prescription numbers, patient-dependent codes, treatment dates, active ingredients, trade names, National Pharmaceutical Product Interference (NAPPI) codes, prescriber and provider specialities, and prescriber postal codes were among the data fields in the database used for this study. 

### 2.2. Statistical Analysis

The data cleaning and analysis were conducted using the statistical software R^®^ version 4.3.1. The chi-squared test was the hypothesis test used for comparisons among distinct groups pertaining to categorical variables. Participants were grouped into age bands corresponding to the time when they were scheduled to receive specific vaccines following the private immunisation schedule. Given that children do not often come on the exact date scheduled for the vaccine, we allowed for a two-week delay in receiving vaccines for participants up to 14 weeks old (6–8 weeks for vaccines due at 6 weeks). For participants aged six months and above, we considered delays up to one month. To calculate the proportion of children who received a specific vaccine at a given time point, the numerator was the number of children in that age group who received the vaccine, and the denominator was the total number of children within that age group. Statistical significance was ascertained through a two-sided probability threshold of *p* < 0.05. Outcomes exhibiting statistical significance were subjected to further scrutiny for their practical significance. In the context of comparing categorical variables, the assessment of effect size was conducted by calculating Cramér’s V. A value of 0 for Cramér’s V indicates the absence of any association between the two examined groups, irrespective of the dimensions of the population [21]. Conversely, a value of 1 for Cramér’s V signifies a perfect association between the variables under investigation. Notably, if the value for Cramér’s V falls below 0.25, a weak association is discerned, while a Cramér’s V value exceeding 0.75 denotes a strong association. In cases where Cramér’s V lies within the range of 0.25 to 0.75, the association is deemed moderate.

### 2.3. Delineated Categories for Prescriber Specialities and Provider Specialities

The delineated categories for prescribers were as follows:

- General medical practitioners: This classification encompassed health and medical practitioners who were duly registered with the Health Professions Council of South Africa (HPCSA) or the relevant regulatory authority within their respective jurisdictions [22].
- Pharmacotherapists: Under this classification, all pharmacotherapists were included, notably primary care drug therapy pharmacists (PCDTs) registered with the South African Pharmacy Council (SAPC) as PCDT pharmacists. Inclusion necessitated additional qualifications that conferred authorisation for PCDT pharmacists to administer their services within primary healthcare [23].
- Pharmacists: This grouping comprised pharmacists, pharmacy assistants, and interns who were registered with the SAPC. Pharmaco-therapists were explicitly excluded from this category [23].
- Other: This category encapsulated an array of prescriber designations, for example, anaesthesiologists, approved day clinics, cardiologists, paediatricians, clinical haematologists, community dentists, community health providers, dermatologists, diagnostic radiologists, and gastroenterologists, to name a few.

The PBM database contains comprehensive information about the entities engaged in providing pharmaceutical products or medication. These entities were delineated into distinct categories as follows:

- General medical practitioners: This classification encompassed all medical practitioners registered at the time with the Health Professions Council of South Africa (HPCSA), encompassing healthcare providers in South Africa. Furthermore, it encompassed medical practitioners who had registrations with regulatory bodies in other countries, thereby allowing them to distribute and administer pharmaceutical products.
- Pharmacies: This category included all pharmacies that held registrations with the SAPC. It also accommodated pharmacies registered with mandatory regulatory bodies in other countries, irrespective of whether the dispensing personnel consisted of a pharmacist, a pharmacist intern, or a pharmacy assistant [23].
- Other: This grouping comprised a diverse array of healthcare providers, notably cardiologists, diagnostic radiologists, dermatologists, general dentists, and gastroenterologists, to name a few.

### 2.4. Ethical Considerations

Ethics approval for this study was obtained from the Health Research Ethics Committee (HREC) of North-West University (ethics approval number NWU-00179-14-A1-14). A waiver for informed consent from patients was granted by the HREC.

Furthermore, formal permission was granted by the PBM company, allowing access to their data. To safeguard privacy and confidentiality, the data provided by the PBM company underwent anonymisation. This process entailed the removal of all identifying information relating to medical schemes, beneficiaries, and service providers.

Subsequently, the dataset, in the form of a comma-separated values file, was securely stored on the password-protected personal computers of the research team members. Access to these data was contingent upon the execution of a confidentiality agreement, reinforcing the commitment to preserving patient confidentiality.

## 3. Results

### 3.1. Demographic Results

A total of 67,830 children aged two years and younger were included in the database from the PBM company. There was a higher proportion of boy (52%) than girls (48%).

### 3.2. Prescriber Speciality by Year

**Figure 1:**
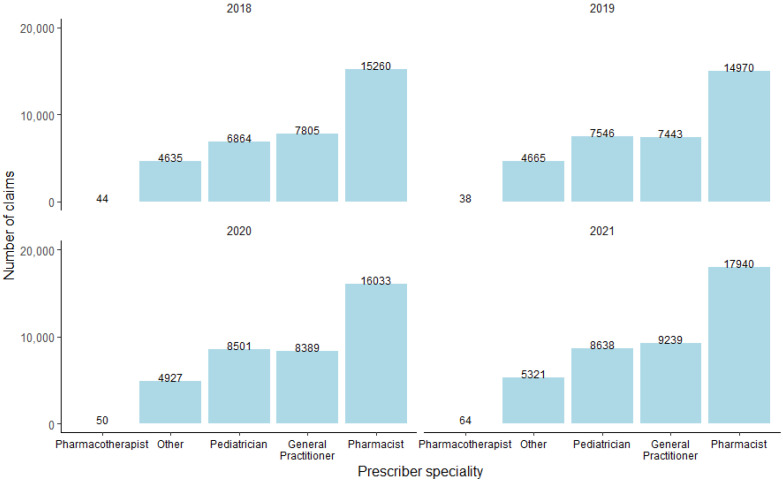
The difference in prescriber specialties and the number of medicine claims before and during COVID-19.

In the context of this investigation, pharmacists were consistently identified as the prescriber category with the highest volume of claims in both study periods, as delineated in Figure 1. In stark contrast, pharmacotherapists were the prescribers contributing the fewest claims throughout the duration of this study. It is of particular note that each identified category of prescribers demonstrated a progressive increase in the number of claims submitted annually over the study period. Figure 1 visualizes the changes in prescriber specialties alongside the corresponding volume of medicinal claims, both before and amidst the COVID-19 pandemic, providing a clear graphical representation of the trends and variations within the data. 

### 3.3. Provider Speciality

**Figure 2:**
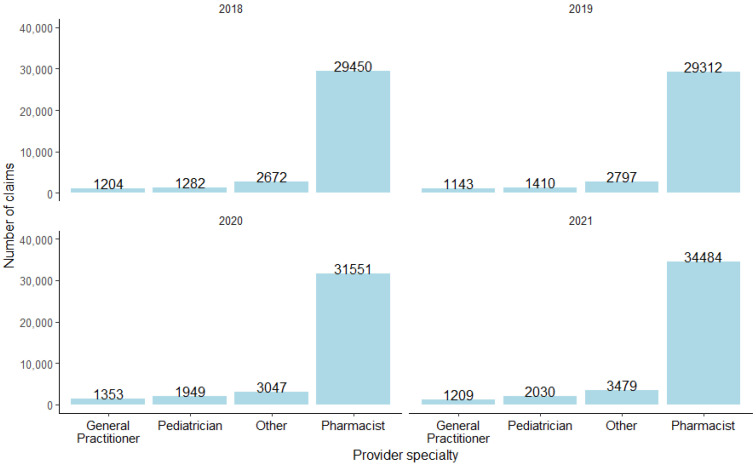
The different provider specialties and the number of medicine claims before and during COVID-19.

From 2018 to 2021, pharmacists were the most frequent healthcare providers of vaccines (Figure 2). 

### 3.4. Vaccines by Province

The prevalence of children who received non-COVID-19 vaccines was higher prior to the onset of the COVID-19 pandemic (60%), as opposed to during the pandemic (55%) (Table 1). This discernible augmentation of pre-COVID prevalence, compared to the phase during the COVID-19 pandemic, was consistently replicated across all provinces and was statistically significant (*p* value < 0.001).

### 3.5. Vaccines in the First Year of Life

Compared to the period before COVID (Table 2), there was a reduction in the proportion of children who received the oral polio vaccine (OPV) birth dose, rotavirus vaccine (RVV) at 10–12 weeks, PCV at 10–12 weeks, measles vaccine (at 6 and 9 months), and flu vaccine. Conversely, there was an increase in the proportion of children who received the other vaccines administered in the first year of life.

### 3.6. Vaccines in the Second Year of Life

A noteworthy elevation in vaccination rates was evident both before and during the COVID-19 periods. Particularly prominent was the substantial increase observed for the measles, hepatitis A, chickenpox, and pentavalent vaccines (Table 3). 

## 4. Discussion

This analysis, aimed at assessing the vaccine coverage patterns before and during the COVID-19 pandemic, found an overall increase in the administration of non-COVID-19 vaccines to children in South Africa’s private healthcare sector. This finding is not consistent with the results of other global studies that aimed to determine the impact of the COVID-19 pandemic on vaccination patterns. A comparative analysis conducted between the years 2019, designated as the pre-pandemic period, and 2021, characterized by the pervasive influence of the COVID-19 pandemic, revealed noteworthy trends in vaccination coverage across distinct global regions. Specifically, the findings indicated a notable decrease in vaccination coverage rates within the Americas by 4%, reflecting a substantial deviation from pre-pandemic levels. Similarly, the Southeast Asia region exhibited a more pronounced decline of 7% in vaccination coverage during the pandemic era, indicative of the heightened challenges in sustaining immunization initiatives amidst the prevailing health crisis. Furthermore, the Eastern Mediterranean region experienced a notable reduction of 3% in vaccination coverage [24]. 

Another consequence of the disruptions precipitated by the COVID-19 pandemic on routine vaccination programs was the substantial rise in the population of zero-dose children. In the African continent, the number of zero-dose children has markedly increased from 7.1 million to 7.7 million, signalling a concerning escalation in vaccine coverage gaps [25]. Conversely, in the Americas, despite a decrease in absolute numbers, a persistent presence of 1.7 million zero-dose children underscores the lingering challenges in achieving universal immunization coverage. Similarly, within the East Asian region, a notable ascent from 1.8 million to 2.3 million zero-dose children highlights the formidable barriers hindering vaccination accessibility and uptake. Meanwhile, in the Southeast Asian region, a notable surge from 2.0 million to 4.1 million zero-dose children emphasises the acute vulnerabilities exacerbated by the pandemic, necessitating urgent and targeted interventions to mitigate the burgeoning risks of vaccine-preventable diseases [25]. 

According to the findings of this study, the most substantial increase in vaccination prevalence from before to during the pandemic manifested within the Limpopo province. The Eastern Cape visibly exhibited the most extensive prevalence before and during the pandemic. The observed increase in numerical values in these particular provinces can plausibly be attributed to the implementation of catch-up initiatives orchestrated by World Vision South Africa and AMREF Germany in 2021 [26]. The primary objective of these programmes was to increase vaccine coverage after the substantial decline encountered during the COVID-19 pandemic [13]. This decline was evident in the statistics, which indicated a decrease from 82% in April 2019 to 61% in April 2020 [10].

There was a significant reduction in measles vaccinations for infants in the first year of life. Specifically, there was a substantial reduction in measles vaccination coverage from 5.6% to 0.7% at six months and from 5.1% to 0.1% at nine months of age. It is unclear why there was such a marked reduction for measles vaccines and not for other vaccines administered at similar time points. These findings align with those the World Health Organization (WHO), revealing that a mere 83% of children across the global spectrum received their inaugural measles vaccine dose, marking the lowest coverage since 2008 [13]. A comparable trend unfolded in the United States, where a decline in measles vaccine uptake materialised due to individual opt-outs for personal reasons [27]. This provides an alternative rationale for this decline in coverage surfaces from the disruptions created by the COVID-19 pandemic, impacting routine immunisation programmes [27,28].

A discernible escalation was noted in the administration of the BCG, RVV, pentavalent, and PCV vaccines for children under one year. Conversely, in the context of vaccines allocated during the second year of life, the proportion of measles vaccinations increased from 22% to 28%. Notably, augmentations in vaccine coverage were also observed for the chickenpox vaccine, hepatitis A vaccine, meningococcal vaccine, and pentavalent vaccine within the demographic of children in their second year of life. This augmented uptake is reasonably attributed to intensified campaigns and endeavours aimed at fortifying immunisation initiatives within the private healthcare sector in 2021 [29,30].

In the context of prescriber classifications, pharmacists consistently maintained their position as the prescriber category associated with the highest count of medicine claims for vaccinations throughout the study period. This delineation underscores the dynamic nature of prescriber activity and its evolution in response to the global health crisis, highlighting the pivotal role of pharmacists in the healthcare continuum during such periods of heightened demand. Within the realm of provider specialty, pharmacists consistently emerged as the foremost contributors across all years under study, exhibiting a notable predominance. These results underscore the pivotal role pharmacists play in vaccine distribution, particularly in the vaccination efforts. One plausible rationale for this finding may be the perception of patients, who have consistently attested to pharmacists’ unparalleled accessibility and readily available provision of healthcare services, a sentiment particularly accentuated amid the exigencies imposed by the COVID-19 pandemic. This observation highlights the pivotal role played by pharmacists in ensuring the accessibility and continuity of healthcare services, particularly in the context of vaccination administration, thus corroborating their enduring prominence within the healthcare landscape [31,32].

Given the observed reduction in claims for certain vaccines in our dataset, it would be helpful to emphasise the importance of childhood vaccination using various communication channels to increase vaccination uptake in South Africa.

There are a few limitations to our findings. It is important to note that the scope of this study is limited to a section of the private healthcare sector of South Africa and does not provide a comprehensive picture of the entire population. Furthermore, this study did not include data on cash purchases of vaccines in the private healthcare sector but relied solely on medicine claim data. Despite these inherent limitations, the findings serve as a foundational assessment of vaccine prescribing trends both before and during the COVID-19 pandemic within a subset of South Africa’s private healthcare sector.

## 5. Conclusions

Immunisation uptake in the private healthcare sector in South Africa increased among children in their second year of life during the COVID-19 pandemic compared to the period before the pandemic. There was some variation for vaccines administered in the first year of life, with increased uptake for some vaccines and decreased uptake for others. Pharmacists were the predominant healthcare provider and prescriber of vaccines throughout the study period.

Given these circumstances, urgent measures are imperative to improve vaccination uptake for all antigens administered to children under two years of age within the private healthcare sector. These efforts must particularly target children who missed vaccine doses during the COVID-19 pandemic. Such initiatives are vital to avert the looming threat of outbreaks of vaccine-preventable diseases.

## Figures and Tables

**Table 1:** The analysis concerning children who were administered non-COVID vaccines, encompassing both the provincial and national dimensions.

Province	Before COVID		During COVID		*p* Value
	Participants (N)	Received Non-COVID Vaccine: n (%)	Participants (N)	Received Non-COVID Vaccine: n (%)	
Eastern Cape	3244	958 (30%)	2880	974 (34%)	<0.001
Free State	3531	1524 (43%)	3245	1537 (47%)	<0.001
Gauteng	20,725	7666 (37%)	18,761	7462 (40%)	<0.001
KwaZulu-Natal	5276	1657 (31%)	4556	1707(37%)	<0.001
Limpopo	3646	1177 (32%)	3825	1486 (39%)	<0.001
Mpumalanga	3491	1281 (37%)	4922	2143 (44%)	<0.001
North West	2224	725 (33%)	2114	798 (38%)	<0.001
Northern Cape	2850	759 (27%)	2696	877 (33%)	<0.001
Western Cape	14,947	2966 (20%)	12,949	2836 (22%)	<0.001
National	41,004	16,329 (40%)	38,977	17,615 (45%)	<0.001

**Table 2:** Non-COVID-19 vaccines administered to children within designated age ranges according to the private immunisation schedule in South Africa before and during the COVID-19 pandemic.

Timing of Vaccine Administration	Age Group Total (n)	Before COVID	During COVID	*p* Value *	Effect Size
At birth	2728	n = 1433	n = 1295		
Bacillus Calmette Guerin (BCG)		52 (3.6%)	59 (4.6%)	0.2	0.023
Oral Polio Vaccine (OPV)		41 (2.9%)	35 (2.7%)	0.8	0.005
At 6 to 8 weeks	13,732	n = 6968	n = 6764		
Oral Polio Vaccine (OPV)		82 (1.2%)	112 (1.7%)	0.017	0.020
Rotavirus Vaccine (RVV)		3835 (55%)	4086 (60%)	<0.001	0.054
Hexavalent ^		4065 (58%)	4330 (64%)	<0.001	0.058
Pneumococcal Conjugate Vaccine (PCV)		3906 (56%)	4157 (61%)	<0.001	0.055
At 10 to 12 weeks	12,217	n = 6264	n = 5953		
Rotavirus Vaccine (RVV)		1432 (23%)	992 (17%)	<0.001	0.078
Hexavalent ^		4061 (65%)	4242 (71%)	<0.001	0.069
Pneumococcal Conjugate Vaccine (PCV)		1755 (28%)	1312 (22%)	<0.001	0.069
At 14 to 16 weeks	12,389	n = 6336	n = 6053		
Rotavirus Vaccine (RVV)		3300 (52%)	3531 (58%)	<0.001	0.063
Hexavalent ^		3820 (60%)	4055 (67%)	<0.001	0.070
Pneumococcal Conjugate vaccine (PCV)		3472 (55%)	3720 (61%)	<0.001	0.067
At 6 to 7 months	23,881	n = 12,496	n = 11,385		
Measles Vaccine		362 (5.6%)	39 (0.7%)	<0.001	0.139
Flu Vaccine		178 (1.4%)	87 (0.8%)	<0.001	0.031
At 9 months	16,109	n = 8178	n = 7931		
Measles Vaccine		418 (5.1%)	9 (0.1%)	<0.001	0.156
Pneumococcal Conjugate Vaccine (PCV)		1947 (24%)	2833 (36%)	<0.001	0.130
Meningococcal Vaccination		1325 (16%)	1585 (20%)	<0.001	0.049

* Pearson’s chi-squared test. ^ Hexavalent containing diphtheria, tetanus, pertussis, polio, hepatitis B, and Haemophilus influenza type b.

**Table 3:** A compilation of non-COVID-19 vaccines administered to children during the second year of their life, both preceding and amidst the COVID-19 pandemic.

Timing of Vaccine Administration	Age Group Total (n)	Before COVID	During COVID	*p* Value	Effect Size
12 to 15 months	32,448	n = 16,560	n = 15,888		
Measles		3695 (22%)	4398 (28%)	<0.001	0.062
Chickenpox		716 (4.3%)	1375 (8.7%)	<0.001	0.088
Hepatitis A		862 (5.2%)	2143 (13%)	<0.001	0.143
Meningococcal vaccine		2167 (13%)	2439 (15%)	<0.001	0.032
18 months	23,436	n = 11,919	n = 11,517		
Hepatitis A		391 (3.3%)	1225 (11%)	<0.001	0.145
Pentavalent		2826 (24%)	3369 (29%)	<0.001	0.063

## Data Availability

The authors do not have permission to share the data.
